# 4210 Da and 1866 Da polypeptides as potential biomarkers of liver disease progression in hepatitis B virus patients

**DOI:** 10.1038/s41598-021-96581-4

**Published:** 2021-08-20

**Authors:** Yuanyuan Ren, Lei Yang, Man Li, Jian Wang, Huimin Yan, Ning Ma, Wenxuan Liu, Liqin Wang, Xia Gao, Ping Gao, Tao Li, Dianwu Liu

**Affiliations:** 1 Hebei Key Laboratory of Environment and Human Health, Department of Epidemiology and Statistics, School of Public Health, Hebei Medical University, Shijiazhuang, 050017 China; 2 Department of Food Quality and Safety, College of Food Science and Biology, Hebei University of Science and Technology, Shijiazhuang, 050018 China; 3 Clinical Research Center, Shijiazhuang Fifth Hospital, Shijiazhuang, 050021 China; 4 Department of Epidemiology, Hebei North University, Zhangjiakou, 075000 China

**Keywords:** Cancer, Biomarkers, Diseases

## Abstract

HBV infection is recognized as a serious global health problem, and hepatitis B virus infection is a complicated chronic disease leading to liver cirrhosis (LC) and hepatocellular carcinoma (HCC). New biochemical serum markers could be used to advance the diagnosis and prognosis of HBV-associated liver diseases during the progression of chronic hepatitis B into cirrhosis and HCC. We determined whether the 4210 Da and 1866 Da polypeptides are serum metabolite biomarkers of hepatopathy with hepatitis B virus. A total of 570 subjects were divided into five groups: healthy controls, those with natural clearance, and patients with CHB, LC, and HCC. The 1866 Da and 4210 Da polypeptides were measured by Clin-ToF II MALDI-TOF–MS. There were significant differences in 4210 Da and 1866 Da levels among the five groups (*P* < 0.001). For the differential diagnosis of CHB from normal liver, the areas under the receiver operating characteristic (ROC) curve of 4210 Da and 1866 Da and their combination via logistic regression were 0.961, 0.849 and 0.967. For the differential diagnosis of LC from CHB, the areas under the ROC curve were 0.695, 0.841 and 0.826. For the differential diagnosis of HCC from CHB, the areas under the ROC curve were 0.744, 0.710 and 0.761, respectively. For the differential diagnosis of HCC from LC, the areas under the ROC curve of 4210 Da and 1866 Da were 0.580 and 0.654. The positive rate of 1866 Da was 45.5% and 69.0% in AFP-negative HCC patients and that of 4210 Da was 60.6% 58.6% in AFP-negative HCC patients of the study HCC vs. CHB and HCC vs. LC. The 4210 Da and 1866 Da polypeptide levels were positively correlated with HBV DNA levels (*P* < 0.001, r = 0.269; *P* < 0.001, r = 0.285). The 4210 Da and 1866 Da polypeptides had good diagnostic value for the occurrence and progression of HBV-related chronic hepatitis, liver cirrhosis and hepatocellular carcinoma and could serve to accurately guide treatment management and predict clinical outcomes.

## Introduction

HBV infection is recognized as a serious global health problem, and more than 257 million people are chronically infected with hepatitis B virus (HBV)^1^. Chronic hepatitis B (CHB) infection is a complex chronic disease that leads to cirrhosis and hepatocellular carcinoma (HCC)^2^. It has been reported that over 880,000 annual deaths are the result of hepatitis B-related outcomes such as liver cirrhosis and HCC^1^. The evolution of chronic hepatitis B into liver cirrhosis and HCC is very complicated. HBV infection is an important etiology of HCC and contributes to over 50% of total HCC cases worldwide^3,4^. Hepatocellular carcinoma (HCC), a common fatal pernicious illness, is the third leading cause of cancer-related death worldwide^5,6^. Therefore, the early diagnosis, early detection and timely intervention of high-risk patients with HBV are of great significance to improve the prognosis of liver cirrhosis and HCC^7,8^. AFP is one of the tumor serum markers commonly used in HCC screening. However, the sensitivity of AFP is reported to range from 39–64%^9,10^. Hence, the development of novel serum biomarkers that better reflect the progression of HBV-related chronic hepatitis, liver cirrhosis and HCC is meaningful.

Biomarkers usually refer to disease-related proteins or biochemical indicators, which are used for clinical diagnosis or monitoring disease activities and have certain guiding significance for prognosis, development and molecular targeted therapy^11,12^. Proteomics can be applied to study biomarkers. Matrix-assisted laser desorption/ionization time-of-flight mass spectrometry (MALDI-TOF-MS) is a promising technology because of its rapid characterization of various biomolecules, so it can be used to analyze biomarkers^13–15^.

MALDI-TOF-MS was applied to detect the protein expression profile of HBV-related chronic liver disease and to search for potential serum markers. In previous studies, it was found that the 4210 Da protein was expressed differently in HBV-related chronic liver disease groups and was confirmed as eukaryotic peptide-releasing factor GTP-binding subunit 3b (eRF3b)^16^. However, there was an absence of healthy controls in the previous studies. Then, the 1866 Da protein, which was also expressed differently among different HBV-related chronic liver disease groups, was later identified as complement C3f-des-arginine (DRC3f)^17^. In this study, we measured the expression levels of these two polypeptides in the sera of healthy controls (HCs), individuals with natural clearance (NC) of hepatitis B, CHB, and patients with LC and HCC. Then, we studied the diagnostic significance of the 4210 Da and 1866 Da proteins in HBV-related chronic hepatopathy, cirrhosis and liver cancer, especially in HCC patients in the early stages. The relationship between the expression of 4210 Da and 1866 Da and HBV DNA was also studied.

## Results

### Demographic and laboratory characteristics of the subjects

The clinicopathological characteristics of patients and control subjects are shown in Table 1. There were no significant differences in age or sex among the study groups. Compared with the control group, there were significant differences in most laboratory parameters in the patient group (all *P* > 0.05). In addition, there were significant differences in ALT, AST, TBIL, DBIL, ALB and HBV DNA between the HBV patient groups with and without HCC (all *P* < 0.05).

**Table 1:** Clinical characteristics of the subjects in each set.

Variable	HC (*N* = 130)	NC (*N* = 50)		CHB (*N* = 130)	LC (*N* = 130)	HCC (*N* = 130)	*P* value
Age (years, mean ± SD)	55.9 ± 12.5	57.30 ± 12.0		55.3 ± 9.2	55.8 ± 11.2	57.1 ± 7.5	0.357
Sex (male/female)	92/38	31/19		97/33	98/32	102/28	0.209
ALT (U/l), *M* (*QR*)	–	–		78.00 (95.00)	39.00 (29.00)	40.00 (33.00)	< 0.001
AST (U/l, *M* (*QR*)	–	–		59.00 (73.00)	45.50 (41.00)	47.00 (82.00)	0.011
TBIL (μmol/l), *M* (*QR*)	–		–	17.00 (29.00)	30.55 (30.00)	30.0 (38.00)	< 0.001
DBIL (μmol/l), *M* (*QR*)	–		–	7.00 (20.00)	12.50 (15.00)	11.00 (20.00)	< 0.001
ALB (g/l, *M* (*QR*)	–		–	41.20 (10.00)	34.50 (8.00)	36.20 (9.00)	< 0.001
HBV DNA (log_10_ IU/ml)	–		–	5.68 (3.51)	3.08 (3.28)	2.74 (1.50)	< 0.001
AFP(ng/ml)				6.80 (23.60)	7.36 (37.60)	53.00 (320.60)	< 0.001

Data are presented as the mean ± SD (mean ± standard deviation).

*M (QR)* median (quartile interval), *AST* aspartate aminotransferase, *ALT* alanine aminotransferase, *TBIL* total bilirubin, *DBIL* direct bilirubin, *ALB* albumin, *HBV* hepatitis B virus, *HC* healthy control, *NC* natural clearance, *CHB* chronic hepatitis B, *LC* liver cirrhosis, *HCC* hepatocellular carcinoma.

### Levels of serum 4210 Da and 1866 Da polypeptides

**Figure 1:**
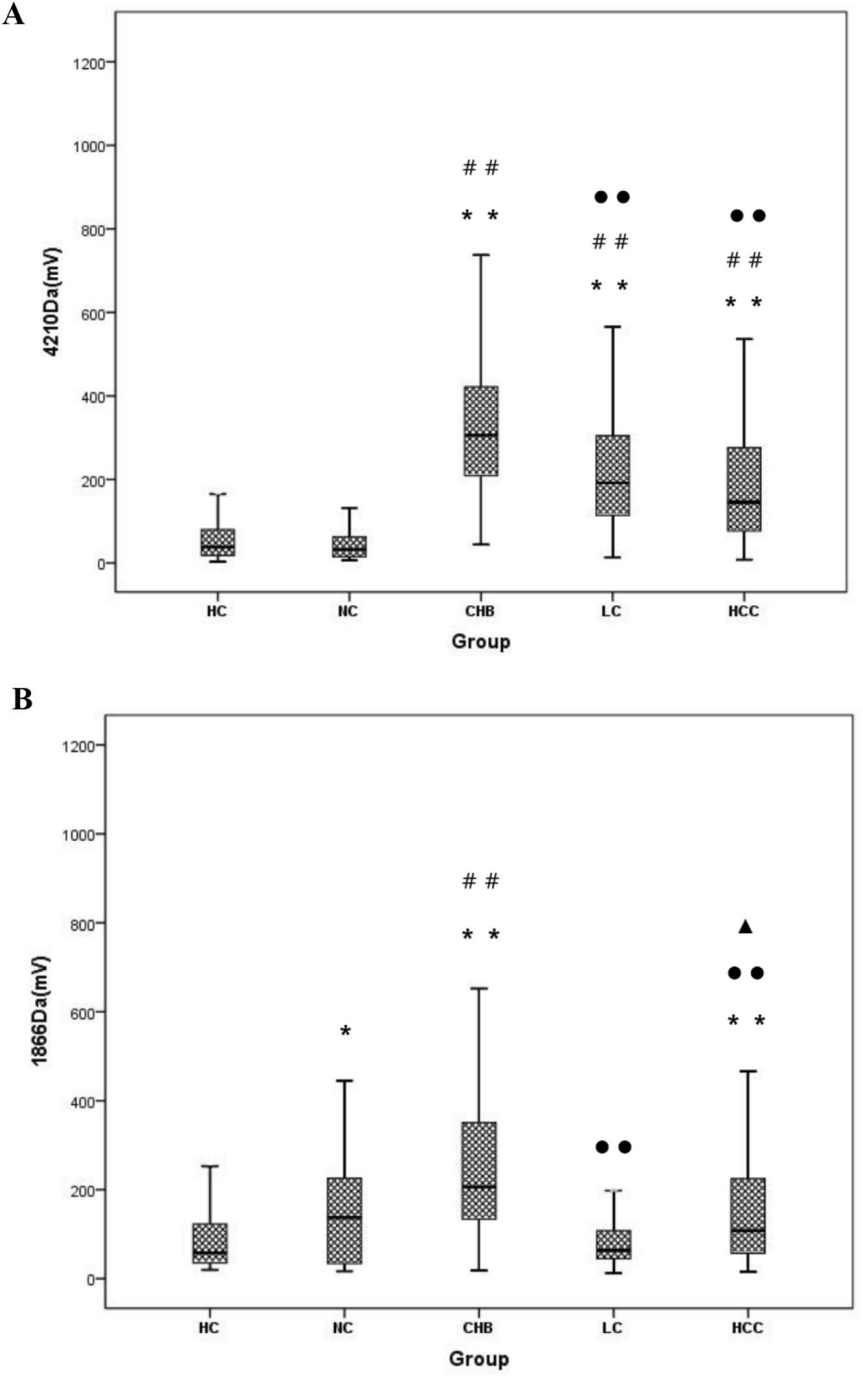
Box plots of the detection results of the 4210 Da and 1866 Da polypeptides in the sera of patients from each group. The top and bottom of the boxes represent the first and third quartiles, respectively, and the horizontal lines across the boxes represent the median values. (**A**) 4210 Da polypeptide (*χ*^2^ = 267.3, *P* < 0.001). (**B**) 1866 Da polypeptide (*χ*^2^ = 127.4, *P* < 0.001). *HC* healthy control, *NC* natural clearance, *CHB* chronic hepatitis B, *LC* liver cirrhosis, *HCC* hepatocellular carcinoma. **P < 0.01 vs. HC; *P < 0.05 vs. HC; ^##^*P* < 0.01 vs. NC; ^●●^*P* < 0.01 vs. CHB; ^▲^*P* < 0.05 vs. LC.

The levels of serum 4210 Da and 1866 Da polypeptides in each group (HC, NC, CHB, LC, and HCC) were measured by MALDI-TOF MS. As shown in Fig. 1A, there were significant differences in the levels of the 4210 Da polypeptide among the five groups (*χ*^2^ = 267.3, *P* < 0.001). The levels of serum 4210 Da were evidently higher in the CHB, LC and HCC groups than in the HC and NC groups (all *P* < 0.001). The levels of serum 4210 Da were obviously higher in the CHB group than in the LC and HCC groups (all *P* < 0.001). There were no significant differences in 4210 Da levels between the LC and HCC groups (*P* = 0.632). As shown in Fig. 1B, there were also significant differences in the levels of the 1866 Da polypeptide among the five groups (*χ*^2^ = 127.4, *P* < 0.001). The levels of serum 1866 Da were evidently higher in the NC group than in the HC group (*P* = 0.023) and significantly higher in the CHB and HCC groups than in the HC group (*P* < 0.001). Serum 1866 Da levels were significantly higher in the CHB group than in the NC group (*P* < 0.001). The levels of serum 1866 Da were evidently higher in the CHB group than in the LC and HCC groups (*P* < 0.001). The levels of serum 1866 Da were obviously higher in the HCC group than in the LC group (*P* = 0.001).

### Differentiating powers of the 4210 Da and 1866 Da polypeptides

The differentiating power of 4210 Da and 1866 Da polypeptides expressions were analyzed in patients with CHB from HC, LC from CHB, HCC from CHB, and HCC from LC.

**Figure 2:**
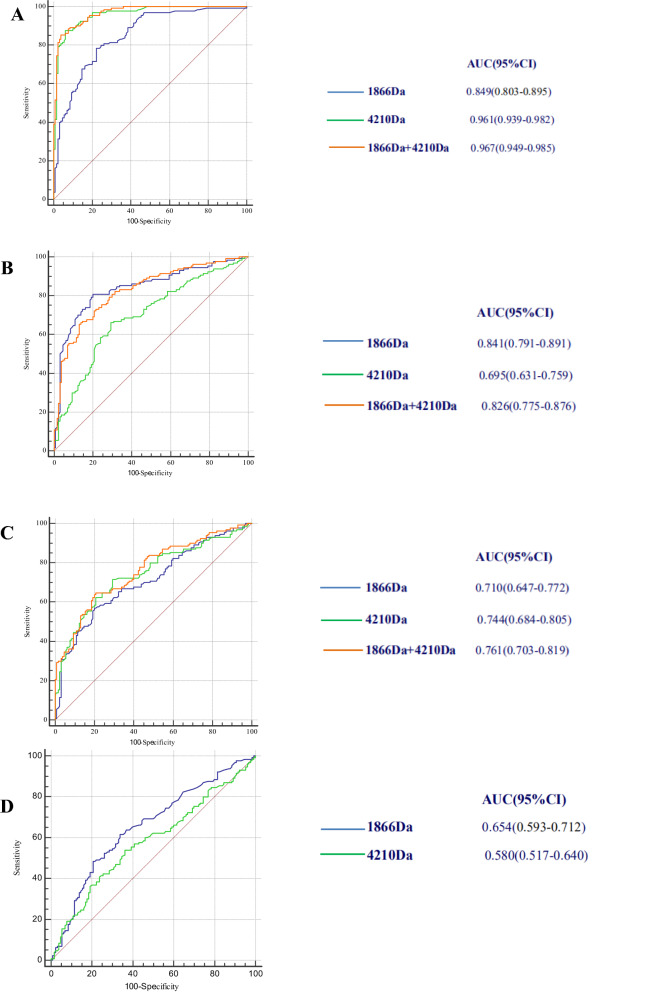
ROC curves of 1866 Da, 4210 Da, and their combination. (**A**) ROC curves of 1866 Da, 4210 Da, and their combination for the differential diagnosis of CHB from normal liver (*P* < 0.001 for 4210 Da vs. 1866 Da, *P* < 0.001 for 1866 Da plus 4210 Da vs. 1866 Da alone, *P* = 0.189 for 1866 Da plus 4210 Da vs. 4210 Da alone). (**B**) ROC curves of 1866 Da, 4210 Da, and their combination for the differential diagnosis of LC from CHB (*P* < 0.001 for 4210 Da vs. 1866 Da, *P* = 0.231 for 1866 Da plus 4210 Da vs. 1866 Da alone, *P* < 0.001 for 1866 Da plus 4210 Da vs. 4210 Da alone). (**C**) ROC curves of 1866 Da, 4210 Da, and their combination for the differential diagnosis of HCC from CHB (p = 0.382 for 4210 Da vs. 1866 Da, p = 0.040 for 1866 Da plus 4210 Da vs. 1866 Da alone, p = 0.384 for 1866 Da plus 4210 Da vs. 4210 Da alone). (**D**) ROC curves of 1866 Da, 4210 Da for the differential diagnosis of HCC from LC (p = 0.166 for 4210 Da vs. 1866 Da).

1. The differentiating power of 4210 Da polypeptide expression was evaluated in patients with CHB and HC. ROC curve analysis showed that the 4210 Da polypeptide was useful in differentiating CHB from HC, with an area under the curve (AUC) of 0.961 (95% CI 0.939–0.982, *P* < 0.001; Fig. 2A). Similar analysis indicated that the AUC for the 1866 Da polypeptide was 0.849 (95% CI 0.803–0.895, P < 0.001; vs. 4210 Da, *P* < 0.001, Fig. 2A). The AUC for the combination testing of both 4210 Da and 1866 Da was 0.967 (95% CI 0.949–0.985, *P* < 0.001; 1866 Da plus 4210 Da vs. 1866 Da alone, *P* < 0.001; 1866 Da plus 4210 Da vs. 4210 Da alone, *P* = 0.189; Fig. 2A), as calculated with the logistic regression model equation: Logit (Y) = − 4.150 + 0.005 × 1866 Da + 0.022 × 4210 Da (*χ*^2^ = 235.47, *P* < 0.001). The diagnostic critical values of 4210 Da and 1866 Da and their combination for CHB from HC via logistic regression were 139.00 mV, 129.50 mV and 0.660, respectively. The sensitivity, specificity, Youden index, accuracy of judgment, positive predictive value, negative predictive value, positive likelihood ratio and negative likelihood ratio of the diagnostic indicators (4210 Da, 1866 Da and their combination via logistic regression) were as follows: 87.7%, 78.5% and 85.4%; 93.8%, 77.7% and 96.2%; 81.5%, 56.2% and 81.5%; 90.8%, 78.1% and 90.8%; 93.4%, 77.9% and 95.7%; 88.4%, 78.3% and 86.8%; 14.145, 3.520 and 22.474; and 0.131, 0.277 and 0.152 (Table 2). These results showed that the combination of the two polypeptides had better diagnostic value than either alone for CHB from normal liver.
2. Then, the differentiation power of the 4210 Da polypeptide level was analyzed in patients with LC from CHB. The AUC for the 4210 Da polypeptide was 0.695 (95% CI 0.631–0.759, *P* < 0.001; Fig. 2B). Similar analysis indicated that the AUC for the 1866 Da polypeptide was 0.841 (95% CI 0.791–0.891, *P* < 0.001; vs. 4210 Da, *P* < 0.001; Fig. 2B). ROC curve analysis showed that the AUC of the combination testing of both 4210 Da and 1866 Da was 0.826 (95% CI 0.775–0.876, *P* < 0.001; Fig. 2B), which did not improve the diagnostic accuracy for LC from CHB compared with 1866 Da alone (1866 Da plus 4210 Da vs. 1866 Da alone, *P* = 0.231; 1866 Da plus 4210 Da vs. 4210 Da alone, *P* < 0.001; Fig. 2B). The logistic regression model equation was as follows: Logit (Y) = − 2.028 + 0.007 × 1866 Da + 0.003 × 4210 Da (*χ*^2^ = 79.95, *P* < 0.001). For LC from CHB, the diagnostic critical values of 4210 Da and 1866 Da and their combination via logistic regression were 232.50 mV, 124.50 mV and 0.347, respectively. LC was diagnosed when the detection value was lower than the critical value. The sensitivity, specificity, Youden index, accuracy of judgment, positive predictive value, negative predictive value, positive likelihood ratio and negative likelihood ratio of the diagnostic indicators (4210 Da, 1866 Da and their combination via logistic regression) were as listed in Table 2. These results showed that the combination of the two polypeptides had better diagnostic value for CHB from cirrhosis than either alone.
3. Next, the differentiating power of 4210 Da and 1866 Da polypeptide expression was evaluated in patients with HCC and CHB. The AUC for the 4210 Da polypeptide was 0.744 (95% CI 0.684–0.805, *P* < 0.001; Fig. 2C). Similar analysis indicated that the AUC for the 1866 Da polypeptide was 0.710 (95% CI 0.647–0.772, *P* < 0.001; vs. 4210 Da *P* = 0.382; Fig. 2C). ROC curve analysis showed that the AUC of the combination testing of both 4210 Da and 1866 Da was 0.761 (95% CI 0.703–0.819, *P* < 0.001), which increased the diagnostic accuracy for HCC from CHB (1866 Da plus 4210 Da vs. 1866 Da alone, *P* = 0.040; 1866 Da plus 4210 Da vs. 4210 Da alone, *P* = 0.384; Fig. 2C). The logistic regression model equation was as follows: Logit (Y) = − 1.730 + 0.003 × 1866 Da + 0.004 × 4210 Da (*χ*^2^ = 53.140, *P* < 0.001). For the differential diagnosis of HCC from CHB, the diagnostic critical values of 4210 Da and 1866 Da and their combination via logistic regression were 233.50 mV, 124.00 mV and 0.439, respectively. HCC was diagnosed when the detection value was lower than the critical value. The sensitivity, specificity, Youden index, accuracy of judgment, positive predictive value, negative predictive value, positive likelihood ratio and negative likelihood ratio of the diagnostic indicators (4210 Da, 1866 Da and their combination via logistic regression) were also listed in Table 2. These results show that the two polypeptides had good diagnostic value for the development of CHB with HCC, and the effect of the combined application was better.
4. Last, the differentiating power of 4210 Da and 1866 Da polypeptide expression was evaluated in patients with HCC from LC. The AUC for the 4210 Da polypeptide was 0.580 (95% CI 0.517–0.640, *P* = 0.026; Fig. 2D). Similar analysis indicated that the AUC for the 1866 Da polypeptide was 0.654 (95% CI 0.593–0.712, *P* < 0.001; vs. 4210 Da, *P* = 0.166; Fig. 2D). For the differential diagnosis of HCC from LC, the diagnostic critical values of 4210 Da and 1866 Da were 152.00 mV, and 80.00 mV respectively. For 4210 Da, HCC was diagnosed when the detection value was lower than the critical value 152.00. For 1866 Da, HCC was diagnosed when the detection value was equal to or higher than the critical value 80.00 mV. The evaluation indexes of the diagnostic effectiveness of 1866 Da and 4210 Da were listed in Table 2.

**Table 2:** Evaluation indexes of the diagnostic effectiveness of 1866 Da, 4210 Da and AFP.

	AUC (95% CI)	Se (%)	Sp (%)	Accuracy (%)	YI (%)	+ PV (%)	− PV (%)	+ LR	− LR
**CHB vs. HC**									
1866 Da	0.849 (0.803–0.895)	78.5	77.7	78.1	56.2	77.9	78.3	3.520	0.277
4210 Da	0.961 (0.939–0.982)	87.7	93.8	90.8	81.5	93.4	88.4	14.145	0.131
1866 Da + 4210 Da	0.967 (0.949–0.985)	85.4	96.2	90.8	81.5	95.7	86.8	22.474	0.152
**LC vs. CHB**									
1866 Da	0.841 (0.791–0.891)	80.8	80.0	80.4	60.8	80.2	80.6	4.040	0.240
4210 Da	0.695 (0.631–0.759)	66.2	70.8	68.5	37.0	69.4	67.7	2.267	0.477
1866 Da + 4210 Da	0.826 (0.775–0.876)	66.9	85.4	76.2	52.3	82.1	72.1	4.582	0.388
**HCC vs. CHB**									
1866 Da	0.710 (0.647–0.772)	56.9	80.0	68.5	36.9	74.0	65.0	2.845	0.539
4210 Da	0.744 (0.684–0.805)	71.5	70.8	71.2	42.3	71.0	71.3	2.449	0.403
1866 Da + 4210 Da	0.761 (0.703–0.819)	64.6	79.2	71.9	43.8	75.6	69.1	3.106	0.447
AFP	0.683 (0.623–0.739)	71.8	52.7	62.3	24.5	60.3	65.1	1.518	0.535
**HCC vs. LC**									
1866 Da	0.654 (0.593–0.712)	61.5	66.2	63.1	27.7	78.4	46.2	1.820	0.582
4210 Da	0.580 (0.517–0.640)	53.9	63.9	57.2	17.8	74.9	40.9	1.493	0.721
AFP	0.716 0.657–0.770	70.8	50.0	63.9	20.8	73.9	46.1	1.416	0.574

*AUC* area under the curve, *Se* sensitivity, *Sp* specificity, *YI* Youden index, *+ PV* positive predictive value, *− PV* negative predictive value, *+ LR* positive likelihood ratio, *− LR* negative likelihood ratio.

### The comparison between AFP and the two polypeptides

The differentiating power of AFP the polypeptides expressions were compared in patients with HCC from CHB, and HCC from LC.

**Figure 3:**
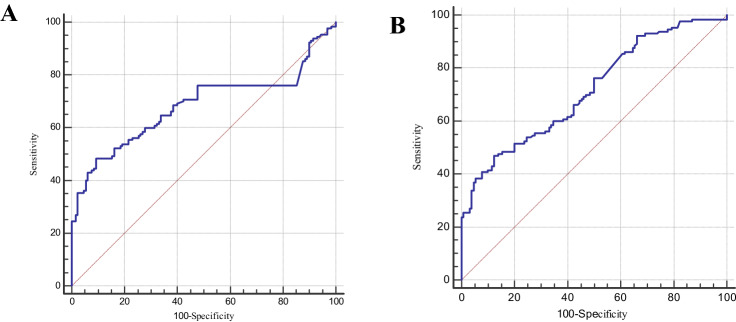
ROC curves of AFP. (**A**) ROC curves of AFP for HCC from CHB (0.683, 95% CI 0.623–0.739, *P* = 0.026) (**B**) ROC curves of of AFP for HCC from LC (0.716, 95% CI 0.657–0.770, *P* < 0.001).

1. The AUC for AFP with HCC from CHB was 0.683 (95% CI 0.623–0.739, *P* = 0.026; Fig. 3A). At the cut-off 7.00 ng/ml, the evaluation indexes of the diagnostic effectiveness of AFP were listed in Table 2. There were no significant differences in the areas under the ROC curve between AFP and the two polypeptides for HCC from CHB (AFP vs. 1866 Da alone, *P* = 0.570; AFP vs. 4210 Da alone *P* = 0.191; AFP vs.1866 Da plus 4210 Da, *P* = 0.093).To evaluate the value of the two polypeptides in AFP-negative patients, the serum levels of 1866 Da and 4210 Da were analyzed in AFP-negative patients with HCC. According to previous research results from HCC patients, when the detection value of serum 1866 Da was less than 124.00 mV or when the detection value of serum 4210 Da was less than 233.50 mV, the patient was diagnosed with HCC. In this study, there were 33 AFP-negative HCC patients. The positive rate of 1866 Da in AFP-negative patients with HCC was 45.5%, and the positive rate of 4210 Da in AFP-negative patients with HCC was 45.5% (Table 3). Therefore, the two peptides had important diagnostic significance in AFP-negative HCC patients (in the study of HCC vs. CHB).
2. The AUC for AFP with HCC from LC was 0.716 (95% CI 0.657–0.770, *P* < 0.001; Fig. 3B). At the cut-off 7.00 ng/ml, the evaluation indexes of the diagnostic effectiveness of AFP were listed in Table 2. There were no significant differences in the areas under the ROC curve between AFP and 1866 Da for HCC from LC (AFP vs. 1866 Da alone, *P* = 0.145; AFP vs. 4210 Da alone *P* = 0.004). According to previous research results for HCC from LC, when the detection value of serum 1866 Da ≥ 80.00 mV or when the detection value of serum 4210 Da was less than 152.00 mV, the patient was diagnosed with HCC. In this study, there were 29 AFP-negative HCC patients. The positive rate of 1866 Da in AFP-negative patients with HCC was 69.0%, and the positive rate of 4210 Da in AFP-negative patients with HCC was 58.6% (Table 4). Therefore, the two peptides also had diagnostic significance in AFP-negative HCC patients (in the study of HCC vs. LC)**.**

**Table 3:** Rates of 1866 Da and 4210 Da in AFP-negative patients with HCC (HCC vs. CHB).

Variable	1866 Da		4210 Da	
	Positive result/total no. in cohort	Positive rate (%)	Positive result/total no. in cohort	Positive rate (%)
AFP negative	15/33	45.5%	20/33	60.6%

AFP negative < 7.00 ng/ml; Positive result for 1866 Da < 124.00 mV; Positive result for 4210 Da < 233.50 mV.

**Table 4:** Rates of 1866 Da and 4210 Da in AFP-negative patients with HCC (HCC vs. LC).

Variable	1866 Da		4210 Da	
	Positive result/total no. in cohort	Positive rate (%)	Positive result/total no. in cohort	Positive rate (%)
AFP negative	20/29	69.0%	17/29	58.6%

AFP negative < 7.00 ng/ml; Positive result for 1866 Da 124.00 ≥ 80.00 mV; Positive result for 4210 Da < 152.00 mV.

### 4210 Da and 1866 Da polypeptide levels correlate significantly with HBV DNA in the serum

**Figure 4:**
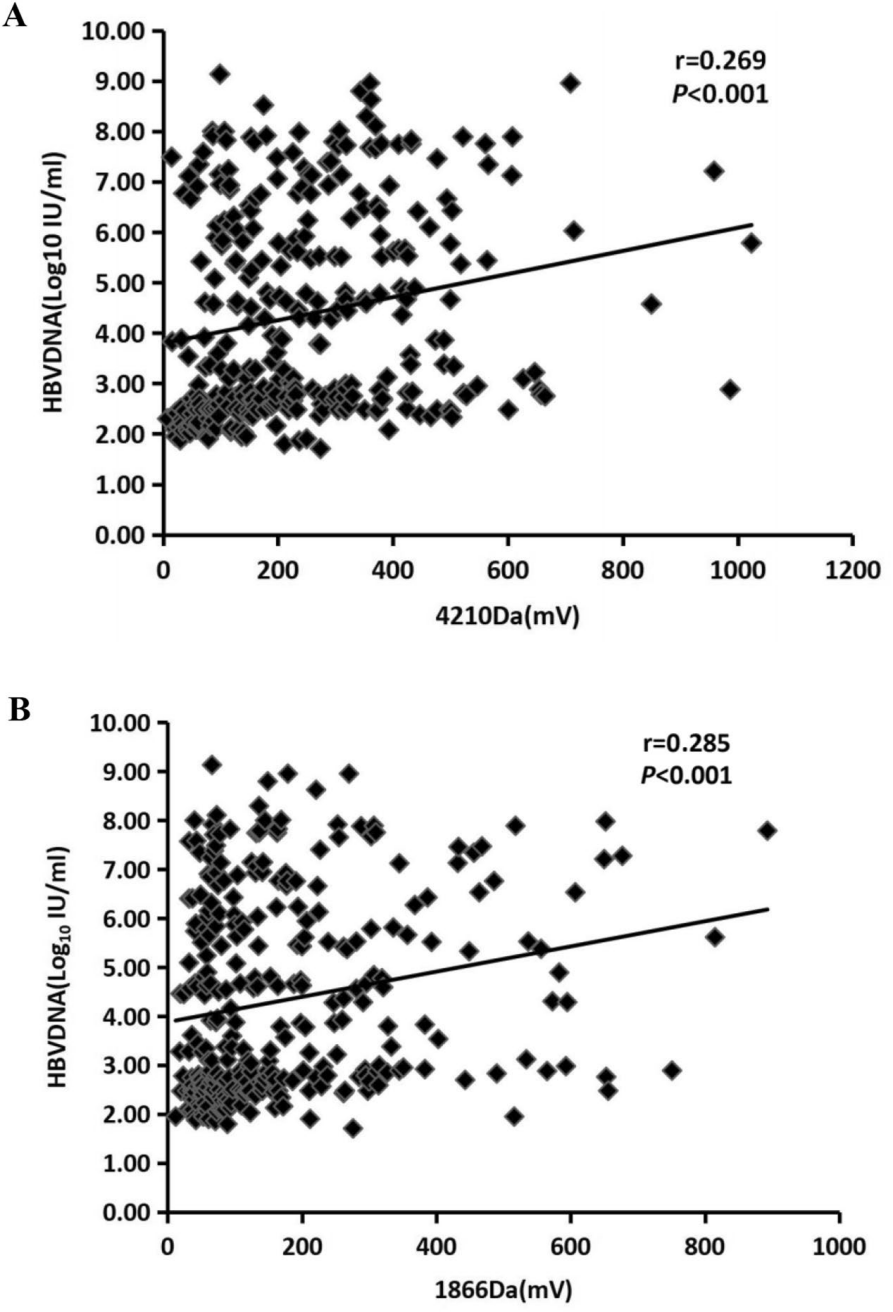
Correlation between serum HBV DNA levels and polypeptide levels in CHB, LC, and HCC patients. (**A**) Correlation between serum HBV DNA levels and 4210 Da polypeptide levels. (**B**) Correlation between serum HBV DNA levels and 1866 Da polypeptide levels.

To better understand the clinical significance of 4210 Da and 1866 Da polypeptide levels in CHB, LC and HCC patients (n = 390), we analyzed the relationship between 4210 and 1866 Da polypeptide levels and HBV DNA levels. Further analysis showed that the 4210 Da polypeptide levels correlated significantly with HBV DNA levels in serum (*P* < 0.001, r = 0.269, Fig. 4A). Spearman linear correlation analysis also revealed that the 1866 Da polypeptide levels were positively correlated with HBV DNA expression levels (*P* < 0.001, r = 0.285, Fig. 4B).

## Discussion

Human serum contains diverse types of peptides. Polypeptides that change due to disease may become valuable biomarkers for the diagnoses of various diseases. Proteomic analyses are very important methods for the analysis and recognition of polypeptides in the serum. The discovery of biomarkers via proteomics has allowed great progress to be made in liver disease^1,4,18–21^ and other diseases^22^. It has been verified that aberrant epigenetic signatures associated with fibrosis and HCC are released into the bloodstream, providing blood-based biomarkers^20^. Biomarkers could be used to advance the diagnosis and prognosis of liver-associated diseases^20^. Diagnosis and remedy during the early stage of chronic hepatitis B can reduce the incidence of liver cirrhosis and cancer. It is important to find biomarkers that can effectively reflect the evolution of HBV-related liver diseases.

On the basis of previous research conducted in our laboratory^16^, in this study, we measured the expression levels of the 4210 Da and 1866 Da polypeptides in the sera of healthy controls, people with natural clearance and patients with hepatitis B-related chronic hepatitis, liver cirrhosis and HCC. The results demonstrated that the 4210 Da and 1866 Da polypeptide levels differed in the five groups of research subjects. The contents of serum 4210 Da and 1866 Da were substantially higher in the CHB group than in the LC and HCC groups. ROC curve analysis demonstrated that serum 4210 Da and 1866 Da had significant diagnostic value for CHB from HC. The AUC for the combination of 4210 Da and 1866 Da was 0.967, with a sensitivity of 85.4%. When LC patients were compared with CHB patients, the AUC for the 1866 Da protein was 0.841. At the cutoff value of 124.50 mV for the 1866 Da protein, the optimal sensitivity was 80.8%. The 4210 Da and 1866 Da proteins also had significant diagnostic value for HCC from CHB. The AUC for the combination of 4210 Da and 1866 Da was 0.761, with a sensitivity of 64.6%. There were no significant differences in the areas under the ROC curve between AFP and the two polypeptides for HCC from CHB and HCC from LC, but the two polypeptides have predictive value for HCC in AFP-negative patients.

Hepatitis B virus infection is one of the major risk factors for liver cancer worldwide. HBV is also an important major risk factor for HCC development through direct and indirect mechanisms^23^. Chronic HBV infection contributes to a repeated cycle of liver damage and regeneration, which promotes tumorigenesis^21^. Most cases of HBV-associated HCC occur in cirrhotic liver disease, present in 70–90% of cases^21^. Approximately 50% of all HCC patients carry hepatitis B virus DNA^24^. The lifetime risk of HCC in chronic HBV carriers is 10–25 times higher than that in individuals not infected with HBV^25^. Regardless of the presence of baseline cirrhosis, the risk of HCC is significantly higher in patients with sufficient inhibition of HBV DNA replication than in patients with inactive CHB, suggesting that HBV has direct and persistent susceptibility^23^.

The mechanism of liver cancer caused by HBV is very complex. The integration of HBV was first considered a random incident^26,27^. Hepatitis B virus DNA, the genomic nucleic acid of the virus, reflects active viral replication and secretion^21^. At present, the relevance between HBV DNA integration and HCC remains unclear^28^. The virus survives through an epigenetic transcription template in the infected liver nucleus, which is called covalently closed circular DNA (cccDNA)^29^. The viral genome has four different overlapping open reading frames to encode the core of the structure and envelope proteins, virus reverse transcriptase and regulatory X protein, which is considered a cancer protein. Some studies have shown that increasing the dynamic rate of HBV DNA integration induces hepatocyte death and proliferation^30^. The direct carcinogenic effects of HBV include the integration of host genomes that induce deletion, translocation, *cis*/*trans* activation, fusion transcripts, universal genomic instability, and pleiotropic functions for viral transcripts (HBx and HBsAg)^23^. The HBV genome integrates into the hepatocellular genome of the host genome and induces not only mutagenesis via direct insertion but also the genomic instability of different cancer-related genes. It has been reported that viral-human promoter-driven transcription and viral transfer fusion occur in humans^28,31^. HBV-bound tumors have a high level of chromosomal alterations. HBV plays an immediate function in hepatic transformation by triggering common and specific carcinogenic pathways^25,32^. The mechanism by which HBV leads to the occurrence and development of liver cancer is very complex, and there is still much to explore.

In this study, the levels of serum 4210 Da and 1866 Da in the CHB group were obviously higher than those in the LC and HCC groups. The amount of HBV DNA in the CHB group was clearly higher than that in the LC and HCC groups, consistent with previous studies^33–36^. There were also some studies in which the amount of HBV DNA in the CHB group was evidently higher than that in the LC group^37–40^. HBV replication levels mirrored by HBV DNA serum titers accompanied by damage to neuroinflammatory tissues and liver inflammation have been identified to be the most significant predictors for the progression of hepatopathy^41^. Thus, HBV DNA may be a biomarker reflecting the transcriptional activity of HBV. After HBV infects human hepatocytes, a large amount of HBV is replicated. Therefore, the level of HBV DNA increases, inducing an immune response and causing immune damage and an inflammatory response. This might lead to the damage and necrosis of hepatocytes and the development of cirrhosis or even liver cancer. Approximately 70–80% of patients with HCC-related HBV have cirrhosis^24^. In addition, up to 20% of hepatitis B virus-driven liver cancer cases occur without cirrhosis^42,43^. With the development of liver cirrhosis and liver cancer, the number of normal hepatocytes might decrease, and the living conditions of hepatitis B virus are limited, which could reduce the replication frequency of HBV and HBV DNA levels in the blood. Generally, patients with hepatitis B-related HCC have a history of liver cirrhosis^24^. In this study, liver cirrhosis and HCC existed at the same time, possibly because there was no significant difference in virus content between the liver cirrhosis group and the HCC group.

Further studies showed a significant positive correlation between the 4210 Da polypeptide and the HBV DNA level in the serum (*P* < 0.001, r = 0.269, Fig. 2A). The 1866 Da polypeptide was also positively correlated with the level of HBV DNA in the serum (*P* < 0.001, r = 0.285, Fig. 2B). These results show that these two peptides may be important markers of HBV replication. The level of serum HBV DNA is currently the most relevant marker of HBV replication and is a strong predictor of liver disease progression toward liver cirrhosis and HCC^4^.

In our previous work, the 4210 Da polypeptide was confirmed to be part of eRF3b, a GTP-binding subunit of the eukaryotic peptide chain release factor. eRF3b is also a guanosine triphosphate-binding subunit of the eukaryotic peptide chain release factor known as eukaryotic release factor (eRF)^16^. In mammals, the genes encoding eRF3b and eRF3a are structurally homologous and encoded by two different genes, GSPT2 and GSPT1, which are located on the X chromosome and 16 chromosome in humans, respectively^16,44^. Comparison of the 5′ noncoding sequences of GSPT1 and GSPT2 revealed a potential promoter element in the 5′ noncoding region of GSPT1 that may be responsible for the transcription of GSPT2^45^. Some studies have shown that eRF3a is the main factor of mammalian translation termination and that eRF3b can replace eRF3a via a similar function^46^. ERF3 is also related to cell cycle regulation, the cytoskeleton and tumorigenesis^47,48^. eRF3b/GSPT2 is expressed in hepatic tissue. The relative expression of GSPT2/18S rRNA in patients with chronic hepatitis B is higher than that in patients with LC or HCC^16^, consistent with the expression of 4210 Da in the serum in this study. Previous studies have shown that eRF3b could influence the cell cycle of HepG2 cells and affect the phosphorylation state of 4E-BP1 at Ser65^16^. These results demonstrated that eRF3b decreases the protein and mRNA expression levels of connective tissue factors, the pro‑fibrogenic factor collagen I and α-smooth muscle actin (SMA) stimulated by TGF-β^49^. One study showed that the GSPT2-rs974285 polymorphism was not significantly associated with HBV susceptibility, spontaneous recovery or HBV-related diseases^50^. The functions and mechanisms of eRF3b/GSPT2 and 4210 Da in liver disease are not clear at present and are worth further study.

The 1866 Da polypeptide was identified as complement C3f des-arginine (DRC3f). Our previous research showed that fresh s or filtered sera containing DRC3f as well as the synthesized DRC3f and C3f peptides could stimulate the proliferation of QSG-7701 human hepatic cells and that DRC3f could decrease the expression of TGFβ1 and COLI in hepatic cells^17^. Another study showed that DRC3f was associated with vascular involvement and disease activity in systemic sclerosis (SSC). The synthetic polypeptides of C3f and DRC3f enhanced the proliferation of microvascular endothelial cells^51^. One study demonstrated that the area under the ROC curves of complement C3f and fibrin peptide A were the highest in patients with nonalcoholic fatty liver disease and normal controls^52^. Another study on hepatitis C virus (HCV) showed that serum polypeptides, such as C3f-dR, had predictive value for SVR (sustained virological response) to PEG IFN-a/RBV (pegylated interferon-a plus ribavirin) and that the complements may be involved in HCV elimination^53^. Complement C3f fragments were strongly correlated with the levels of MRD and could be valuable for MRD assessments in the clinic, which are beneficial for diagnosing therapeutic conditions for acute leukemia (AL)^54^. In this study, the 1866 Da polypeptide was found to be related to the replication of HBV DNA.

In summary, our study showed that the combination of the 4210 Da and 1866 Da polypeptides had better diagnostic value in the occurrence and development of hepatitis B-related chronic hepatitis, liver cirrhosis and hepatocellular carcinoma, especially HCC in alpha-fetoprotein (AFP)-negative patients, than either alone. Novel serum markers that are representative of the transcriptional and replicative activity of HBV in the liver are necessary and will serve to accurately guide treatment and predict clinical outcomes^1^. However, the roles of the 4210 Da and 1866 Da polypeptides in the pathologic mechanisms of HBV-related liver diseases need to be further studied in the future.

## Materials and methods

### Participants

From January 2016 to June 2018, 570 unrelated participants (130 healthy controls, 50 with natural clearance of HBV, 130 patients with CHB, 130 patients with HBV-related LC and 130 patients with HBV-related HCC) were recruited from the Fifth Hospital of Shijiazhuang City.

Healthy controls were free of HBsAg (hepatitis B surface antigen), anti-HBs (antibody to hepatitis B surface antigen), anti-HBc (hepatitis B virus core antibody) and other HBV biomarkers, as assessed by biochemical parameter and routine blood examinations. Individuals with natural HBV clearance were defined as positive for anti-HBs and anti-HBc but negative for HBsAg. CHB, LC and HCC patients were defined as positive for HBsAg, positive for anti-HBc and positive for anti-HBe or HBeAg for at least 6 months. Individuals with CHB were defined as positive for HBV DNA and rising ALT or AST levels once or more during the period of liver injury histopathologically diagnosed by ultrasonography or laboratory examinations. LC was diagnosed via clinical results, radiological manifestations, laboratory features or cirrhotic pathology from liver biopsy^55,56^. Clinical manifestations mainly included ascites, spontaneous bacterial peritonitis, varices and hepatic encephalopathy. Radiological manifestations mainly included splenomegaly, portal vein dilation, hepatatrophia and varices. Laboratory features mainly included hyperbilirubinemia, a low platelet count, a low white blood cell count, a prolonged prothrombin time and hypoalbuminemia. HCC was defined via pathology and/or an elevation in blood alpha-fetoprotein (> 400 ng/ml) with additional imaging by MRI (magnetic resonance imaging), CT (computed tomography) or ultrasonography^57,58^. Lesions/hepatic focal masses > 2 cm were identified by imaging methods wherein characteristic contrast enhancement features were observed in the arterial phase with venous washout on either MRI or CT. Biopsy was performed for focal liver masses with atypical imaging findings or those detected in noncirrhotic livers. Patients who had HIV, alcoholic hepatic disease, or an antinuclear antibody titer greater than 1:160 with suspected autoimmune diseases were excluded from this study.

The study protocol conformed to the ethical guidelines of the 1975 Declaration of Helsinki and was approved by the Hebei Medical University Ethics Committee.

We confirm that informed consent was obtained from all subjects, and all subjects were over 18 years old (“Supplementary information”).

### Data collection

The following data were collected: baseline information (age, sex, and HBV vaccine [yes/no]), historical illness (family history, course and condition of the illness, with or without other disorders), the results of serological and biochemical testes (HBV DNA, ALT (alanine aminotransferase), AST (aspartate aminotransferase), TBIL (serum total bilirubin), DBIL (serum direct bilirubin), and ALB (albumin) levels).

### Serum polypeptide analysis by MALDI-TOF MS

All serum samples were stored at − 80 °C until analysis. All serum samples were extracted with MB-WCX (weak cation exchange magnetic beads) according to the instructions provided in the magnetic bead kit (Bioyong Technology Company, Beijing, China). Magnetic bead extraction of serum peptides was performed as follows. First, the magnetic bead kit was removed from the refrigerator at 4 °C and manually turned upside down to mix the beads. Two hundred microliters of beads were added to eight consecutive rows of sample tubes on the plate. Ten microliters of magnetic beads, 95 µl of magnetic bead binding buffer, and 10 µl of serum samples were added in turn and slowly pipetted up and down. The mixture was kept at room temperature for 5 min. Second, after the sample tube was placed on a magnetic bead separator for 1 min, the supernatant was removed. Then, 100 µl of magnetic bead cleaning solution buffer was added and mixed thoroughly. After washing two times and removing the supernatant, 10 µl of magnetic bead eluent was added and mixed thoroughly, avoiding air bubbles. The mixture was incubated for 5 min to allow the beads and eluate to float evenly. Third, the sample was placed on a magnetic bead separator and allowed to stand for 1 min to completely separate the magnetic beads from the suspension. The supernatant was then placed in a labeled 0.2 ml sample tube. The peptide eluate was used for mass spectrometry analysis^16,59^.

Next, 1 μl of sample was placed on the target plate. After drying, 1 μl of matrix solution (CHCA, Sigma, 5 mg/ml) was added to the sample. After drying, the sample was detected via MALDI-TOF-MS (Clin-ToF II, Bioyong Technology Company). MALDI-TOF-MS experiments were performed using a linear model with ten laser shots at 70 mV laser power. The peak m/z intensities or values were determined in the range of 1000–10,000 Da^16^.

### Statistical analysis

Continuous variables with a normal distribution are described as the mean and standard deviation (mean ± SD). The comparison of mean values between groups was conducted by one-way analysis of variance (ANOVA) or the Kruskal–Wallis H rank-sum test according to the homogeneity of variance. Continuous data with an abnormal distribution are described as the median and interquartile range (M (QR)). The Kruskal–Wallis test was used to compare multiple groups of abnormally distributed data. The count data were analyzed by the χ^2^ test. ROC curve analysis was performed by using IBM SPSS 21.0 and MedCalc 15.2.2 statistical software. The optimum cutoff value for diagnosis was investigated by maximizing the sum of sensitivity and specificity (The Youden Index was the max)^6,10^.

Likelihood ratio test was used for fitting the degree of the logistic regression model, and the Wald test was used for estimating the regression parameters. The area under the ROC curve was compared by the Z-test. HBV DNA levels were logarithmically transformed. The correlations between 4210 Da (1866 Da) and HBV DNA levels in the serum were analyzed by Fisher’s exact test or Pearson’s χ^2^ test. The combined markers (4210 Da and 1866 Da) were estimated with binary logistic regression, and the values of these functions were used as one marker subjected to ROC curve analysis.

Statistical analyses were performed using IBM SPSS version 21.0 (SPSS Inc., Chicago, IL, USA) or MedCalc version 15.2 (MedCalc Software, Broekstraat, Mariakerke, Belgium). All tests were two-sided, with a significance threshold of *P* < 0.05.

## Supplementary Information

Supplementary Information.
