# Supplementary material for: 4210 Da and 1866 Da polypeptides as potential biomarkers of liver disease progression in hepatitis B virus patients
Source: Sci Rep. 2021 Aug 20;11:16982. doi: 10.1038/s41598-021-96581-4 (PMC8379215; doi:10.1038/s41598-021-96581-4)
Supplement: Supplementary file 1 — Supplementary Information. [file 41598_2021_96581_MOESM1_ESM.docx]

**Supplementary File**

1.The relations of level of 4210Da and 1866Da Polypeptides and clinicopathological parameters (ALT AST, TBIL, DBIL, and ALB)

Spearman linear correlation analysis revealed that 1866 Da polypeptide level was significantly positively correlated with ALT and ALB, and significantly negatively correlated with TBIL and DBIL. 4210 Da polypeptide level was significantly positively correlated with ALT and AST (Fig. 1).


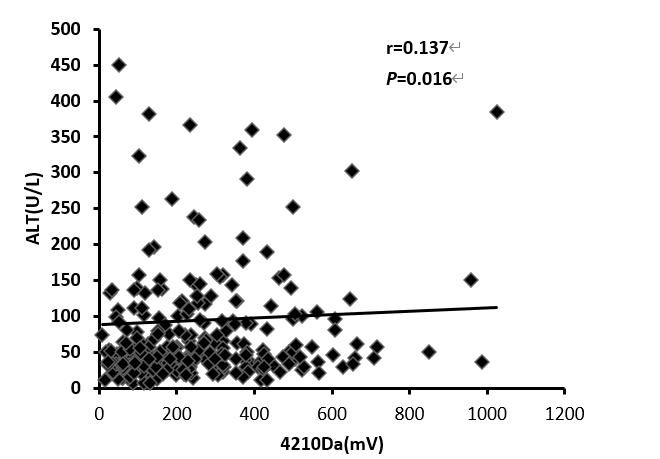

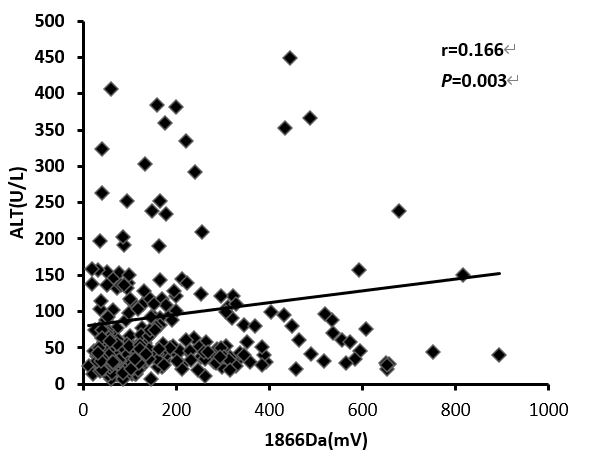


**A**

**B**

**D**

**C**


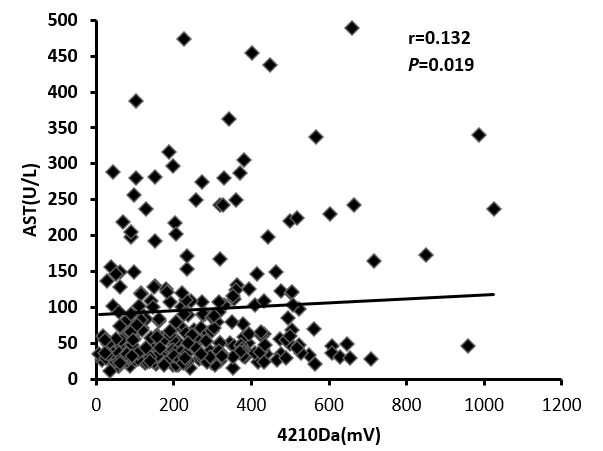

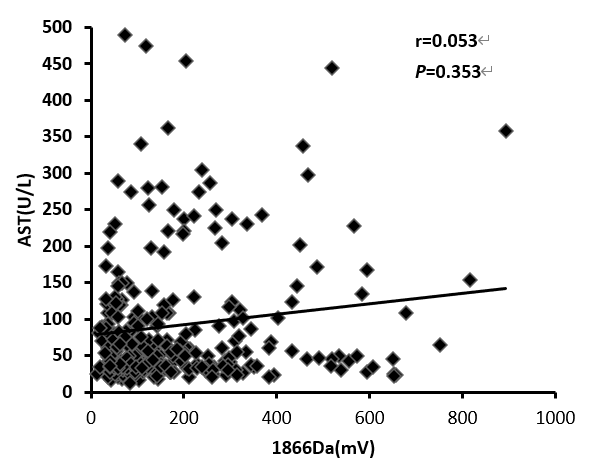


**E**

**F**


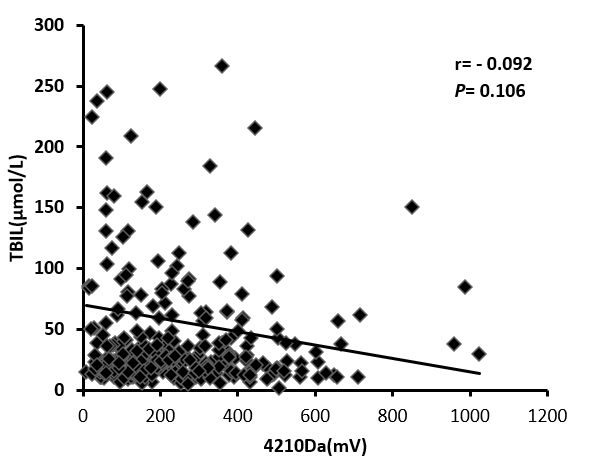

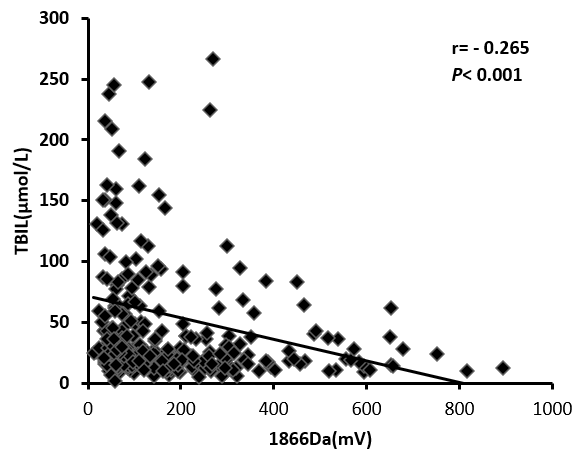


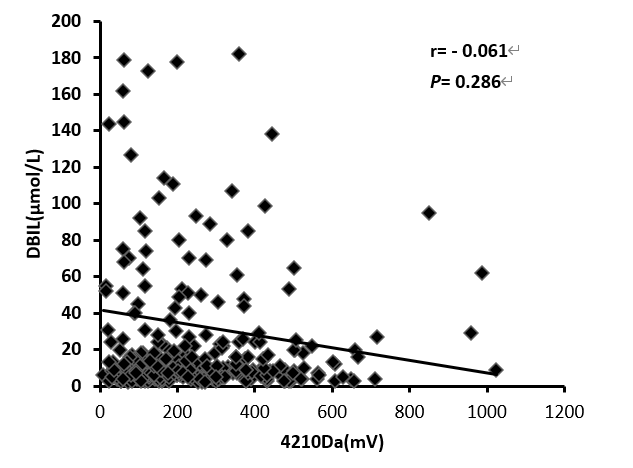

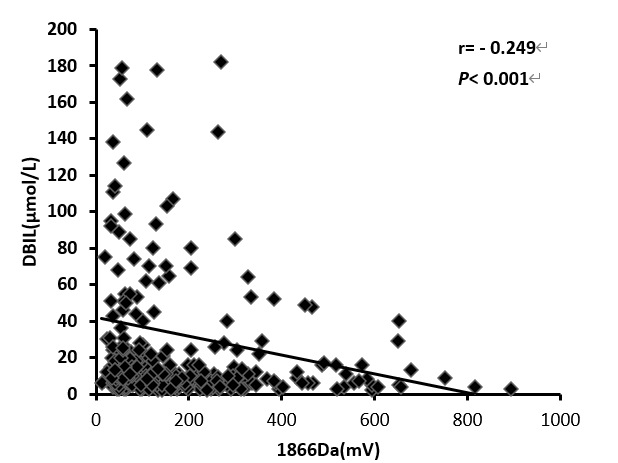


**G**

**H**

**I**


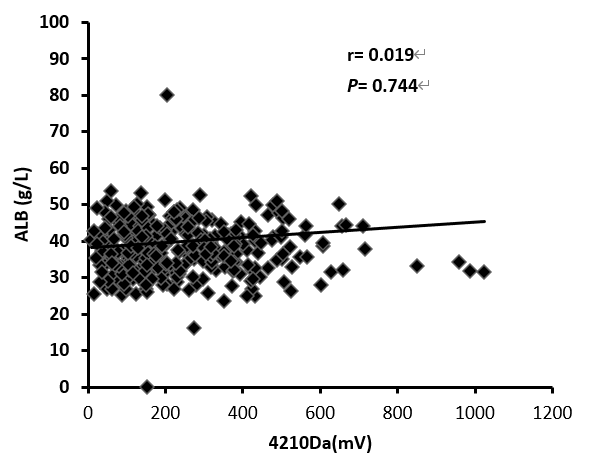

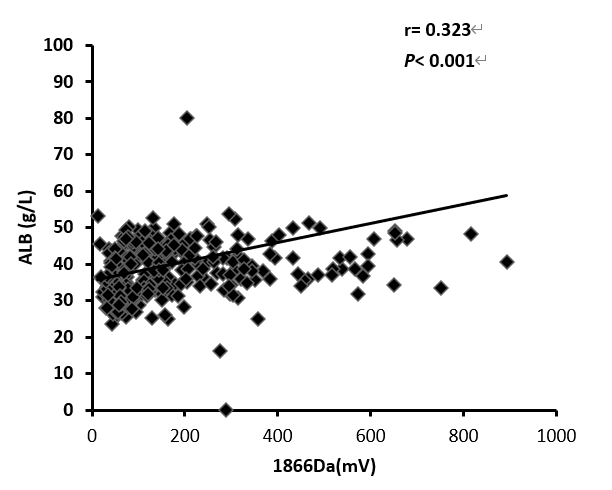


**J**

Fig. 1 Correlation between serum clinicopathological parameters and the two polypeptide levels in CHB, LC, and HCC patients. (A) Correlation between ALT and 4210 Da.(B) Correlation between ALT and 1866 Da. (C) Correlation between AST and 4210 Da.(D) Correlation between AST and 1866 Da. (E) Correlation between TBIL and 4210 Da.(F) Correlation between TBIL and 1866 Da. (G) Correlation between DBIL and 4210 Da.(H) Correlation between DBIL and 1866 Da. (I) Correlation between ALB and 4210 Da.(J) Correlation between ALB and 1866 Da.

**2.Analyzed 4210Da and 1866Da Polypeptides by χ2 test counting variables as positive and negative**

**(1)differentiating CHB from HC**

**1) 4210Da**

Table 1 Diagnostic results of 4210Da polypeptide for CHB from HC

| 4210Da | Clinical diagnosis results | | Total |
| --- | --- | --- | --- |
|  | CHB (Positive) | HC(Negative) |  |
| CHB(Positive) | 114 | 8 | 122 |
| HC(Negative) | 16 | 122 | 138 |
| Total | 130 | 130 | 260 |

Abbreviations: Positive result of 4210Da≥139.00 mV

**2) 1866Da**

Table 2 Diagnostic results of 1866Da polypeptide for CHB from HC

| 1866Da | Clinical diagnosis results | | Total |
| --- | --- | --- | --- |
|  | CHB (Positive) | HC(Negative) |  |
| CHB(Positive) | 102 | 29 | 131 |
| HC(Negative) | 28 | 101 | 129 |
| Total | 130 | 130 | 260 |

Abbreviations: Positive result of 1866Da≥129.50 mV

**3)4210Da+1866Da**

Table 3 Diagnostic results of two polypeptides with logistic model for CHB from HC

| 1866Da +4210Da | Clinical diagnosis results | | Total |
| --- | --- | --- | --- |
|  | CHB (Positive) | HC(Negative) |  |
| CHB(Positive) | 111 | 5 | 116 |
| HC(Negative) | 19 | 125 | 144 |
| Total | 130 | 130 | 260 |

Abbreviations: Positive result of 1866Da +4210Da≥0.660

**(2)differentiating LC from CHB**

**1) 4210Da**

Table 4 Diagnostic results of 4210Da polypeptide for LC from CHB

| 4210Da | Clinical diagnosis results | | Total |
| --- | --- | --- | --- |
|  | LC (Positive) | CHB(Negative) |  |
| LC(Positive) | 86 | 38 | 124 |
| CHB(Negative) | 44 | 92 | 136 |
| Total | 130 | 130 | 260 |

Abbreviations: Positive result of 4210Da<232.50 mV

**2) 1866Da**

Table 5 Diagnostic results of 1866Da polypeptide for LC from CHB

| 1866Da | Clinical diagnosis results | | Total |
| --- | --- | --- | --- |
|  | LC (Positive) | CHB(Negative) |  |
| LC(Positive) | 105 | 26 | 131 |
| CHB(Negative) | 25 | 104 | 129 |
| Total | 130 | 130 | 260 |

Abbreviations: Positive result of 1866Da<124.50 mV

**3)4210Da+1866Da**

Table 6 Diagnostic results of two polypeptides with logistic model for LC from CHB

| 1866Da +4210Da | Clinical diagnosis results | | Total |
| --- | --- | --- | --- |
|  | LC (Positive) | CHB(Negative) |  |
| LC(Positive) | 87 | 19 | 106 |
| CHB(Negative) | 43 | 111 | 154 |
| Total | 130 | 130 | 260 |

Abbreviations: Positive result of 1866Da +4210Da < 0.347

**(3)differentiating HCC from CHB**

**1) 4210Da**

Table 7 Diagnostic results of 4210Da polypeptide for HCC from CHB

| 4210Da | Clinical diagnosis results | | Total |
| --- | --- | --- | --- |
|  | HCC (Positive) | CHB(Negative) |  |
| HCC (Positive) | 93 | 38 | 131 |
| CHB(Negative) | 37 | 92 | 129 |
| Total | 130 | 130 | 260 |

Abbreviations: Positive result of 4210Da<233.50 mV

**2) 1866Da**

Table 8 Diagnostic results of 1866Da polypeptide for HCC from CHB

| 1866Da | Clinical diagnosis results | | Total |
| --- | --- | --- | --- |
|  | HCC (Positive) | CHB(Negative) |  |
| HCC (Positive) | 74 | 26 | 100 |
| CHB(Negative) | 56 | 104 | 160 |
| Total | 130 | 130 | 260 |

Abbreviations: Positive result of 1866Da<124.00 mV

**3)4210Da+1866Da**

Table 9 Diagnostic results of two polypeptides with logistic model

for HCC from CHB

| 1866Da +4210Da | Clinical diagnosis results | | Total |
| --- | --- | --- | --- |
|  | HCC (Positive) | CHB(Negative) |  |
| HCC (Positive) | 84 | 27 | 111 |
| CHB(Negative) | 46 | 103 | 149 |
| Total | 130 | 130 | 260 |

Abbreviations: Positive result of 1866Da +4210Da < 0.439
